# Climate change and food safety—not just a hot topic!

**DOI:** 10.1093/sumbio/qvaf019

**Published:** 2025-09-26

**Authors:** Catherine Rees, John Threlfall

**Affiliations:** School of Biosciences, University of Nottingham, Sutton Bonington Campus, Loughborough, Leicestershire, LE12 5RD, United Kingdom; Executive Committee, Applied Microbiology International, Tregawne Cottages, Bodmin, Cornwall, PL30 5NR, United Kingdom

## Abstract

The impact of climate change on food safety is an evolving area of science. Examples of outbreaks of food and water-related infection possibly related to climate change are reviewed here along with other emerging issues. For instance, increasing sea temperatures are contributing to an increase in *Vibrio spp*. in the aquatic environment and rising cases of foodborne infections and as well resulting in new patterns of fish-related diseases in consumers, such as Ciguatera fish poisoning. Warmer and wetter climactic conditions are also contributing to an increase in mycotoxin production and there is a growing concern that the drive towards more sustainable food supplies and novel alternative protein sources may lead to the emergence of novel threats. Future areas of possible concern (‘*unknown unknowns’*) are climactic conditions driving the appearance of highly virulent foodborne pathogens, increased threats due to increases in noxious seaweed in coastal areas in the Caribbean and Mexico, and dust storms spreading fungal and bacterial pathogens over vast areas globally. To ensure public safety in the future these new threats need to be recognized, and appropriate mitigation measures put in place by the food industry—which may require a re-evaluation of many established HACCP (Hazard Analysis and Critical Control Points) plans.

**Sustainability Statement**

This perspective considers the impact of changes in weather patterns related to climate change on food safety. By collating existing knowledge about outbreaks of disease attributed to adverse weather events, we aim to inform discussion of future challenges that may impact on human health (SDG 3). The issues highlighted underpin the development of new food safety policies and food production processes to reduce the risk of foodborne infections in a changing world. Ensuring safe food also supports SDG 2 (food security).

## Introduction

When we think of the impact of climate change, thoughts go to headlines about heatwaves and wildfires. Data show that the extreme temperatures driving these events are becoming more common; for instance, the three warmest years on record for Europe have all occurred since 2020 (Copernicus Climate Change Service). Similarly, temperatures in the USA for July in 2024 were 1°C above average, with fires affecting almost 4.75 million acres, which was nearly 1 million above the 10-year YTD (Year to Date) average of 3.8 million acres (NIFC 2024). These effects are being seen worldwide; it is reported that 47% of global land area was affected by extreme drought between 2013 and 2022 which was an increase of 19% over a comparable 9-year period between 1951 and 1960. Continuing this trend, in 2024 this increased to 48%, which was 2.5 times higher than levels reported in 2020 (Romanello et al. 2023, 2024). With such large areas of land being affected by either drought or fire damage, it is not surprising that impacts are seen on food production.

Climate change is also driving a pattern of other extreme weather events, such as increased rainfall, catastrophic storms and flooding (Met Office 2024) all of which also have a negative impact on food production. Globally, a 61% increase was seen in the number of days of extreme rainfall in the period 2014–23 compared to 1961–90, thereby increasing the risk of flooding, infectious disease spread and water contamination (Guzman et al. 2016, Romanello et al. 2024).

Besides the obvious concern about the effects of these events on food supply, there is also a concern that these extreme weather events may have a more immediate impact on food safety. For instance, heavy rainfall and flooding can lead to contamination of water and food supplies with faecal pathogens from sewers (Levy et al. 2016, Geremew et al. 2024), increasing risks of foodborne and waterborne diseases such as cholera and salmonellosis (Schwartz et al. 2006, Zellweger et al. 2017, Taseen et al. 2022). In addition, heavy rainfall increases the risk of transfer of zoonotic pathogens onto crops (see section Introduction).

It is not just water quality that can have a direct impact on food safety; foodborne diseases such as salmonellosis and campylobacteriosis are known to be influenced by temperature (Semenza and Paz 2021, Damtew et al. 2024, Balta et al. 2024), thus contributing to higher disease burden and associated healthcare costs, particularly in socioeconomically disadvantaged regions. As global temperatures are predicted to continue to rise, with increasing intensity, frequency, and duration of high temperature periods, it is predictable that an increase in levels of foodborne disease will be seen.

This perspective highlights areas of concern already identified as having a negative effect on food safety, and also those events that may be expected to occur if the rate of climate change continues to increase, particularly in relation to increases in temperature and rainfall.

## Examples of outbreaks of food and water-related infection possibly related to climate change

### Heavy rainfall and flooding

Climate-change associated hazards have been described as having the potential to activate cascading risk pathways for food production with a sequence of secondary, causally connected events. For example, cascading risks associated with heavy precipitation followed by flooding of animal environments may lead to contamination of crops and cause food-borne outbreaks of zoonotic diseases. Higher pathogen loads are frequently detected in floodwater after rainstorms and extreme weather events have been associated with outbreaks of gastrointestinal illness (Mulder et al. 2019, Ethan et al. 2024, Haley, 2024),

The section below describes some key events, both ‘historical’ (pre-2020) and ‘recent’ (post-2020), and provides some examples of incidents and outbreaks of infection that associated with climate change-associated weather events. The common theme in these examples is contamination of water resulting in food contamination—particularly ‘ready to eat (RTE)’ food, resulting in infections by a range of pathogenic microorganisms (see Table 1).

**Table 1.** Examples of outbreaks of infection linked to severe weather events

Date	Event	Details of event	Citation(s)
2001	Increase in GI infections following contact with floodwater, American mid-west.	Severe flooding in the midwest USA. Association between gastrointestinal symptoms and contact with floodwater observed, particularly in children. Thought to be first prospective observation of increase in endemic gastrointestinal symptoms following a flood. Evidence that severe climatic events can result in increase in endemic incidence of gastrointestinal symptoms, even in countries with good sanitary network.	Wade et al. (2004)
2005	Hurricanes Katrina and Rita, USA result in increase in GI infections.	New microbial communities detected in sediments contaminated with raw sewage following hurricanes including DNA sequences associated with pathogens: (1) Infections reported in relief workers and public after Hurricane Rita; 882 skin or wound infections; 1304 acute respiratory infections; 233 cases of diarrhoea; 470 other infectious diseases. (2) In the week after Hurricane Katrina ∼240 000 persons evacuated to a sports and convention complex; substantial numbers of adults and children with symptoms of acute gastroenteritis; 18% with acute symptoms. Norovirus detected in 50% of stool samples by PCR.	Amaral-Zettler et al. (2008), Morantz (2005), CDC (2023)
2011	Drought results in contamination of crops leading to international STEC *Escherichia coli* O104:H4 outbreak.	*∼*4000 cases of *E. coli* O104:H4 (54 deaths) across 5 countries in 2011. Suspect vehicle of infection were fenugreek seeds produced in Egypt. Attributed to fertilization of crop with human sewage following drought in Rive Nile.	Karch et al. (2012)
2014–20	Flood water contamination of crops result in Cyclospora outbreak(s), Mexico.	Series of outbreaks of Cyclosporiasis (foodborne diarrheal illness caused by *Cyclospora cayetanensis*) affecting > 1000 travellers to luxury hotels in Mexico since 2014. Association with cilantro (coriander) grown in water contaminated with human sewage, linked to flooding events.	Nichols et al. (2015), Almeria and Santin (2024)
1995–2018	Contamination of irrigation water; STEC *E. coli* outbreaks, UK.	Six outbreak investigations involving ready-to-eat salad products identified outbreak strain in animal faeces near the crop growing fields; in two cases also found in irrigation water on the farms, providing a likely route of contamination.	Kintz et al. (2019)
2021	Flooding in Belgium, Germany, Luxembourg, and The Netherlands, June/July.	Outbreaks of waterborne infections considered possible; public advised to throw away any food in contact with flood water. People in areas affected by disruptions of sewage water systems considered at risk of increased direct or indirect transmission of several gastrointestinal pathogens, particularly *E. coli*, Norovirus, *Cryptosporidium, Giardia, Campylobacter*, different *Salmonella* enterica serotypes, *Shigella*, Hepatitis A.	ECDC (July 2021)

Four of these examples relate to Shiga toxin-producing *E. coli* (STEC). The gastrointestinal tract of ruminants is the ecological niche of STEC, with cattle and sheep being the main animal reservoirs. While direct contact with colonized animals or their environment can lead to infection, outbreaks of disease more commonly occur following consumption of food or water contaminated with the pathogen. Food items frequently associated with food-borne outbreaks of STEC include raw or undercooked beef or lamb, unpasteurized dairy products and salad vegetables, but rigorous hygiene practices in preparation of these products can limit the risk. It is clear that larger, unpredicted outbreaks of disease have resulted from consumption of fresh produce exposed to rainwater run-off, floodwater, or irrigation water contaminated with animal or human faeces (see Table 1).

It is not only surface water contamination of products that pose a threat. Contamination can also occur during post-harvest washing as highlighted by outbreaks of listeriosis in the US traced to cantaloupe melons when investigations revealed that washing with contaminated water followed by hydrocooling contributed to the outbreak. Subsequent studies found that *Listeria monocytogenes* in wash water can infiltrate intact cantaloupes through the stems/stem scars and spread to the interior, edible parts of the fruit *via* the plant vascular system (Macarisin et al. 2017). *Listeria monocytogenes* is not only a human pathogen but is also an environmental, saprophytic bacterium capable of utilizing plant sugars for growth. So once internalized in the melon—or other fruit—they can grow, and even chilled storage will not prevent this because this psychrotrophic bacterium can readily adapt to growth at low temperatures (Lourenco et al. 2022).

Internalization of nonsaprophytes into plant tissue via water contamination is also a risk. For instance, the other major pathogenic microorganisms associated with the consumption of fruit include *Salmonella, E. coli*, Hepatitis A, and Norovirus. Studies have shown that all these microbes can be internalized into plant structures and that bacterial numbers can increase if cold storage is not maintained. Once internalized pathogens are protected from standard washing and decontamination procedures and can persist long enough to cause infection following ingestion (see Melo and Quintas 2023).

### Higher ambient temperature

As described above, storing food at low temperature to prevent the growth of microbes is a key step in ensuring the safety of foods. Increasing environmental temperatures will require better temperature control in the cold chain (domestic and industrial) to avoid impacting microbiological safety and quality of food (James and James 2010).

Monitoring of zoonoses across Europe shows that the number of reported foodborne outbreaks and cases, hospitalizations and deaths was higher in 2022 than in 2021, with the number of deaths from outbreaks being the highest reported in the EU in the last 10 years. Many of these deaths were attributed to *L. monocytogenes*, and while it is hard to directly link this increase to climate change, the primary source of transmission to humans is the consumption of RTE foods (such as cold smoked salmon, meat and meat products, and dairy products). Interestingly routine surveillance of these products did not detect an increase in levels of food contamination and yet the number of sporadic cases of listeriosis worldwide continues to increase (EFSA 2024, Su et al. 2024). This suggests that improper storage of food post-purchase may be an important contributing factor. In the UK, a number of outbreaks have been associated with pre-prepared sandwiches consumed by patients in hospitals, where storage of the food at room temperature was identified as a key event (see Macleod et al. 2022). Clearly as ambient temperatures rise, there is an increasing concern that low levels of contamination of bacterial pathogens are more likely to reach critical levels in high-risk foods.

Maintaining an effective food cold chain to ensure the quality and safety of perishable food is also an issue for food producers as this must be balanced against costs. In the context of global warming, the sustainability trade-off between waste losses and environmental impact of the food cold chain has become an important topic of discussion. Small food producers (SMEs) are likely to struggle most with the challenges of maintaining the cold chain due to the cost of adopting new technologies and solutions developed primarily for larger food producers (Chen et al. 2022). Since SMEs, rather than microfood producers, are already recognized as being a higher risk of being the source of outbreaks of foodborne disease, further pressure caused by increasing ambient temperatures is not likely to improve the situation.

### Rising sea temperatures

Records show that global sea surface temperatures began rising in the latter half of the 20th century and continue to rise at an average rate of 0.25°C per decade, with a temperature rise or 0.5°C–2.5°C being recorded in most parts of the world (USEPA 2024). Studies have investigated the effect of rising sea temperature on marine microbial populations and have recorded increased bacterial activity associated with increasing temperature (Heneghan et al. 2024). Changes in population structure have also been noted due to the loss of less thermotolerant organisms, causing minor components of the original bacterial population to become more dominant (Marsh 2024, Hutchins and Fu 2017).

Historical samples can provide good evidence for these changes; the analysis of archived formalin-preserved samples collected between 1958 and 2011 from the North Atlantic and North Sea found that the relative abundance of *Vibrio* species, including human pathogens, in nine areas showed correlation with increasing sea surface temperatures (up to ∼1.5°C over 54 years). Comparison with epidemiological data for the same regions revealed that this increase coincided with higher reports of environmentally-acquired *Vibrio* infections in human populations (Vezzuli et al. 2016). A recent review of risk factors associated with the consumption of shellfish in the EU indicate that increasing sea temperature was a key factor driving change in *Vibrio* abundance, resulting in increasing levels in seafood. A survey of infections caused by four species associated with seafood consumption [*Vibrio alginolyticus*, nontoxigenic *Vibrio cholerae* (non-O1/non-O139), *Vibrio parahaemolyticus*, and *Vibrio vulnificus*] in EU countries bordering the Baltic Sea found a concomitant increase in *Vibrio spp*. abundances, infection rates (94% causing disfunction of the GI tract) and deaths across the region. Sweden and Germany had the highest number of cases and the increase in *V. alginolyticus* and *V. parahaemolyticus* infections positively correlated with increasing sea surface temperature (Gyraitė et al. 2024). Mirroring this—at the time of writing—the US public have again been alerted to an increased risk of *V. vulnificus* infections (NBC News Aug 2025), with the CDC noting increasing water temperatures and extreme weather events associated with climate change as significant factors (CDC HAN 2023).

Increasing sea water temperatures may also induce stress responses that drive adaptation/evolution of marine bacteria (Ben-Haim et al. 2003, Cohen et al. 2018). For instance, cell stress due to prolonged incubation at 30°C in an environment closely mimicking their sea microcosm caused up-regulation of many virulence genes of the marine invertebrate pathogen *Vibrio harveyi*, potentially increasing its pathogenicity (Montánchez et al. 2019). Similarly, a large study of strains of *V. parahaemolyticus* isolated from shellfish imported into China from Asia, Europe, North America, South America, and Oceania, detected an increase mutation rate in the thermolabile haemolysin gene (*tlh*). The rate of change correlated with increasing global average temperatures, and many of the mutations detected were predicted to increase the heat stability of the toxin's binding capacity (Zhang et al. 2023). In another example, changes in the pattern of human infections caused by *Streptococcus agalactiae* associated with consumption of raw, freshwater fish has been attributed to the occurrence of adaptive mutations that correlate with increases in the temperature of waters in fish farms and increased losses due to fish disease, which also threatens food security (Lusiastuti et al. 2024).

Foodborne disease can also be caused by contamination of food with naturally occurring toxins produced by fungi and plankton. Increases in the presence of these toxins and/or their appearance in new geographical locations has been linked to climate change in some cases. An example is Ciguatera fish poisoning (CFP) caused by eating reef fish contaminated with ciguatoxins produced by marine dinoflagellates (primarily *Gambierdiscus* and *Fukuyoa;* Costa et al. 2023). CFP typically occurs in the Pacific Ocean, Indian Ocean and the Caribbean Sea with up to 500 000 cases occurring per year, making it the most common cause of seafood poisoning. Since 2008, several outbreaks of CFP have occurred in Spain (Canary Islands) and in Portugal (Madeira) with climate change the likely driver of colonization of new areas by the toxin-producing dinoflagellates (Chinain et al. 2021). Hence climate change is already resulting in the emergence of new patterns foodborne disease.

### Changes in agricultural practice and food production due to climate change—the 'known unknowns'

The previous sections focussed on changes we have already seen that can be linked to climate change. There is however a growing concern that the drive towards more sustainable foods may lead to the emergence of new threats.

The range of novel alternative protein sources is ever increasing and diverse and includes cereals, legumes, seeds, nuts, potatoes, mushrooms and even green leaves (see Banach et al. 2023). Their natural microbial populations are very different to those normally associated with meat or dairy protein, therefore novel technologies are needed to generate products with acceptable consumer characteristics (Sha and Xiong 2020). This raises a new challenge because HACCP plans for the production of safe food generally are based on long-term understanding of the microbial risks associated with different starting materials (Weinroth et al. 2018). For instance, a survey of plant-based meat alternatives identified three different members of the *Bacillus cereus* group which all were diarrhoea‐associated strains in 5% of products tested (Barmettler et al. 2025). Hence, testing regimes and advice to consumers about how to store these products may need to change to avoid future outbreaks of disease.

Cultivated insects are also being developed as a novel protein source and these have also been found to be contaminated with *B. cereus* strains capable of causing foodborne infections and therefore appropriate testing regimen will be needed to ensure product safety (Pal et al. 2024). A more unpredictable risk comes from the use of a waste product from this industry (insect exuviae) as a sustainable soil amendment to promote crop growth and health by stimulating naturally occurring, beneficial microbes. Since some of these products also promoted the growth of both *Pseudomonas* and *Burkholderiaceae* species, there is a potential risk of contamination of crops with these human pathogens (Wantulla et al. 2023).

Yet another predictable problem is an increase in intoxications caused by mycotoxins produced by filamentous fungi. Temperature, humidity, and water availability are key factors that influence both infection of plants by mycotoxigenic fungi and the synthesis of mycotoxins. Changing weather patterns have already been associated with unexpected levels of *Aspergillus flavus* mycotoxins being detected in crops in Italy and France, and in the future is predicted to significantly affect coffee production due an increase in mycotoxin contamination (Zingales et al. 2022). It is not only changes in weather that are relevant; it is predicted that atmospheric concentrations of CO_2_ will reach levels that can cause some mycotoxigenic fungi to increase mycotoxin production during storage of crops (Medina 2023).

### Final thoughts—the 'unknown unknowns'

Until the 1980s, *Listeria* was primarily considered to be a sporadic, opportunist pathogen rather than a foodborne pathogen. Changes in food production practices at this point (i.e. the development of cook-chill products and the rise in popularity of RTE foods) resulted in large outbreaks, and now foodborne listeriosis is recognized as a major threat by the food industry. This illustrates how innovation or changes in food production practices can have unpredictable consequences.

Looking ahead, it is probable that new threats will emerge for which we yet have no evidence to make predictions. Bacteria are adaptive organisms, able to exchange large amounts of genetic information. The selective pressures of a new environmental niche will rapidly see new strains containing combinations of genes emerging that pose new threats. An example of this is the *E. coli* O104: H4 strain responsible for the major international outbreak (Karch et al. 2012); this strain is fundamentally an Enteroaggregative *E. coli* that acquired at least one Shiga toxin (*stx)* gene and resistance to several antibiotics including ESBLs and also a chromosomally-encoded multidrug resistance gene cluster, thus limiting possible treatment options.

Also not yet fully quantified is the impact of climate change on global weather patterns that may change patterns of infection. For example, an outbreak of Aspergillosis in North America in 2020 was attributed to spread of the fungus by a major dust storm originating from the Sahara. Pathogenic bacterial gene sequences were identified in Saharan dust, so these events could also contribute to outbreaks of food poisoning over large areas (Das et al. 2023). Changes in sea currents may also produce unexpected changes in microbial populations; changes in warm currents from the Amazon River entering the Atlantic Ocean off the coast of Brazil have resulted in a massive expansion of *Sargassum* seaweed throughout the Caribbean and has even travelled to the North African coastline. Not only are the seaweed rafts noxious, they support the growth *Vibrio* species shown to carry the zonula occludens toxin, which contributes to chronic diarrhoea and therefore may pose a threat to humans (Mincer et al. 2023).

## Conclusion

So, what can we do to mitigate against these unpredictable events? In the short term the food industry needs to develop new HACCP plans based on an understanding of the microflora of new food products and new tests are required to improve our monitoring of the safety of food products. In the longer term, modelling of weather and environmental conditions may provide early warning of events that drive changes in microbial populations and alert us to new threats. It is, however, clear that ensuring food safety in the face of a changing planet will require concerted international actions—a situation somewhat undermined by the current mood of global isolationist politics?

## Data Availability

No new data were generated or analysed in support of this article.

## References

[bib1] Almeria S, Santin M. Editorial for the Special Issue *Cyclospora cayetanensis* and Cyclosporiasis. Microorganisms. 2024;12:281. 10.3390/microorganisms12020281. 38399685 PMC10893465

[bib2] Amaral-Zettler LA, Rocca JD, Lamontagne MG et al. Changes in microbial community structure in the wake of Hurricanes Katrina and Rita. Environ Sci Technol. 2008;42:9072–8. 10.1021/es801904z. 19174873 PMC2668980

[bib3] Anikeeva O, Hansen A, Varghese B et al. The impact of increasing temperatures due to climate change on infectious diseases. Br Med J. 2024;387:e079343. 39366706 10.1136/bmj-2024-079343

[bib4] Balta I, Lemon J, Murnane C et al. The One Health aspect of climate events with impact on foodborne pathogens transmission. One Health. 2024;19:100926. 10.1016/j.onehlt.2024.100926. 39559751 PMC11570983

[bib5] Banach JL, van der Berg JP, Kleter G et al. Alternative proteins for meat and dairy replacers: food safety and future trends. Crit Rev Food Sci Nutr. 2023;63:11063–80. 10.1080/10408398.2022.2089625. 35757863

[bib61_454_233925] Barmettler K, Waser S and Stephan R. Microbiological Quality of Plant-based Meat-alternative Products Collected at Retail Level in Switzerland. J Food Prot. 2025;88:100402. 10.1016/j.jfp.2024.100402 39542106

[bib6] Ben-Haim Y, Zicherman-Keren M, Rosenberg E. Temperature-regulated bleaching and lysis of the coral *Pocillopora damicornis* by the novel pathogen *Vibrio coralliilyticus*. Appl Environ Microbiol. 2003;69:4236–42. 10.1128/AEM.69.7.4236-4242.2003. 12839805 PMC165124

[bib7] CDC Health Alert Network. Severe Vibrio Vulnificus Infections in the United States Associated with Warming Coastal Waters. CDCHAN-00497. 2023. https://www.cdc.gov/han/2023/han00497.html (August 2025, date last accessed).

[bib8] CDC. Forecasting and outbreak analytics. [Annual Report]. 2023;. https://www.cdc.gov/forecast-outbreak-analytics/pdf/cdc-cfa-annual-report-2023.pdf.

[bib9] Chen Q, Qian J, Yang H et al. Sustainable food cold chain logistics: from microenvironmental monitoring to global impact. Compr Rev Food Sci Food Saf. 2022;21:4189–209. 10.1111/1541-4337.13014. 35904269

[bib10] Chinain M, Gatti CMI, Darius HT et al. Ciguatera poisonings: a global review of occurrences and trends. Harmful Algae. 2021;102:101873. 10.1016/j.hal.2020.101873. 33875186

[bib11] Cohen R, James C, Lee A et al. Marine host-pathogen dynamics: influences of global climate change. Oceanography. 2018;31:182–93. 10.5670/oceanog.2018.201.

[bib12] Copernicus Climate Change Service. European State of the Climate Report. 2023. https://climate.copernicus.eu/esotc/2023.

[bib13] Costa PR, Churro C, Rodrigues SM et al. A 15-year retrospective review of ciguatera in the Madeira Islands (North-East Atlantic, Portugal). Toxins. 2023;15:630. 10.3390/toxins15110630. 37999493 PMC10674775

[bib14] Cunningham N, Jenkins C, Williams S et al. An outbreak of Shiga toxin-producing *Escherichia coli* (STEC) O157:H7 associated with contaminated lettuce and the cascading risks from climate change, the United Kingdom, August to September 2022. Eurosurveillance. 2024;29:36. 10.2807/1560-7917.ES.2024.29.36.2400161. PMC11378517 39239728

[bib15] Damtew YT, Tong M, Varghese BM et al. The impact of temperature on non-typhoidal *Salmonella* and *Campylobacter* infections: an updated systematic review and meta-analysis of epidemiological evidence. EBioMed. 2024;109:105393. 10.1016/j.ebiom.2024.105393. PMC11530612 39418985

[bib16] Das S, McEwen A, Prospero J et al. Respirable metals, bacteria, and fungi during a Saharan-Sahelian dust event in Houston, Texas. Environ Sci Technol. 2023;57:19942–55. 10.1021/acs.est.3c04158. 37943153 PMC10862556

[bib17] ECDC Risk of infectious diseases in flood-affected areas from the European Union. News Story, 20 July 2021. https://www.ecdc.europa.eu/en/news-events/risk-infectious-diseases-flood-affected-areas-european-union.

[bib60_146_232825] EFSA The European Union One Health 2023 Zoonoses report. EFSA J. 2024;22:e9106. 39659847 10.2903/j.efsa.2024.9106 PMC11629028

[bib18] Ethan CJ, Sanchez J, Grant L et al. Relationship between extreme precipitation and acute gastrointestinal illness in Toronto, Ontario, 2012–2022. Epidemiol Infect. 2024;152:e32. 10.1017/S0950268824000207. 38329089 PMC10894888

[bib19] Geremew G, Cumming O, Haddis A et al. Rainfall and temperature influences on childhood diarrhea and the effect modification role of water and sanitation conditions: a systematic review and meta-analysis. Int J Environ Res Public Health. 2024;21:823. 10.3390/ijerph21070823. 39063400 PMC11276699

[bib21] Guzman HB, de Blasio BF, Carlander A et al. Association between heavy precipitation events and waterborne outbreaks in four Nordic countries, 1992–2012. J Water Health. 2016;14:1019–27. 10.2166/wh.2016.071. 27959880

[bib20] Gyraitė G, Kataržytė M, Bučas M et al. Epidemiological and environmental investigation of the ‘big four’ *Vibrio* species, 1994 to 2021: a Baltic Sea retrospective study. Eurosurveillance. 2024;29:2400075. 10.2807/1560-7917.ES.2024.29.32.2400075. 39119721 PMC11312017

[bib22] Haley BM, Sun Y, Jagai JS et al. Association between combined sewer overflow events and gastrointestinal illness in Massachusetts municipalities with and without river-sourced drinking water, 2014–2019. Environ Health Perspect. 2024;132:57008. 10.1289/EHP14213. 38775485 PMC11110654

[bib23] Heneghan RF, Holloway-Brown J, Gasol JM et al. The global distribution and climate resilience of marine heterotrophic prokaryotes. Nat Commun. 2024;15:6943. 10.1038/s41467-024-50635-z. 39138161 PMC11322184

[bib24] Hutchins DA, Fu F. Microorganisms and ocean global change. Nat Microbiol. 2017;2:17058. 10.1038/nmicrobiol.2017.58. 28540925

[bib25] James SJ, James C. The food cold-chain and climate change. Food Res Int. 2010;43:1944–56. 10.1016/j.foodres.2010.02.001.

[bib26] Karch H, Denamur E, Dobrindt U et al. The enemy within us: lessons from the 2011 European *Escherichia coli* O104:H4 outbreak. EMBO Mol Med. 2012;4:841–8. 10.1002/emmm.201201662. 22927122 PMC3491817

[bib27] Kintz E, Byrne L, Jenkins C et al. Outbreaks of shiga toxin-producing *Escherichia coli* linked to sprouted seeds, salad, and leafy greens: a systematic review. J Food Prot. 2019;82:1950–8. 10.4315/0362-028X.JFP-19-014. 31638410

[bib28] Levy K, Woster AP, Goldstein RS et al. Untangling the impacts of climate change on waterborne diseases: a systematic review of relationships between diarrheal diseases and temperature, rainfall, flooding, and drought. Environ Sci Technol. 2016;50:4905–22. 10.1021/acs.est.5b06186. 27058059 PMC5468171

[bib29] Lourenco A, Linke K, Wagner M et al. The saprophytic lifestyle of *Listeria monocytogenes* and entry into the food-processing environment. Front Microbiol. 2022;13:789801. 10.3389/fmicb.2022.789801. 35350628 PMC8957868

[bib30] Lusiastuti AM, Suhermanto A, Hastilestari BR et al. Impact of temperature on the virulence of *Streptococcus agalactiae* in Indonesian aquaculture: a better vaccine design is required. Veterinary World. 2024;17:682–9. 10.14202/vetworld.2024.682-689. 38680157 PMC11045521

[bib31] Macarisin D, Wooten A, De Jesus A et al. Internalization of *Listeria monocytogenes* in cantaloupes during dump tank washing and hydrocooling. Int J Food Microbiol. 2017;257:165–75. 10.1016/j.ijfoodmicro.2017.06.018. 28667935

[bib32] Macleod J, Beeton ML, Blaxland J. An Exploration of *Listeria monocytogenes*, its influence on the UK food industry and future public health strategies. Foods. 2022;11:1456. 10.3390/foods11101456. 35627026 PMC9141670

[bib33] Marsh J. Rising tides and microbes: how climate change is reshaping aquatic life. The Microbiologist. 2024. 9 December 2024. https://www.the-microbiologist.com/features/rising-tides-and-microbes-how-climate-change-is-reshaping-aquatic-life/4620.article.

[bib34] Medina A. Emerging mycotoxin risks due to climate change. What to expect in the coming decade?In: Knowle ME et al. (eds.), Present Knowledge in Food Safety. Academic Press, 2023, 309–14.

[bib35] Melo J, Quintas C. Minimally processed fruits as vehicles for foodborne pathogens. AIMS Microbiol. 2023;9:1–19. 10.3934/microbiol.2023001. 36891538 PMC9988415

[bib36] Met Office (UK's National Meteorological Service). Effects of climate change. https://www.metoffice.gov.uk/weather/climate-change/effects-of-climate-change (Jan 2025, date last accessed).

[bib37] Mincer TJ, Bos RP, Zettler ER et al. Sargasso Sea Vibrio bacteria: underexplored potential pathovars in a perturbed habitat. Water Res. 2023;242:120033. 10.1016/j.watres.2023.120033. 37244770

[bib38] Montánchez I, Ogayar E, Plágaro AH et al. Analysis of *Vibrio harveyi* adaptation in sea water microcosms at elevated temperature provides insights into the putative mechanisms of its persistence and spread in the time of global warming. Sci Rep. 2019;9:289. 10.1038/s41598-018-36483-0. 30670759 PMC6343004

[bib39] Morantz C. CDC Reports on illnesses in hurricane Katrina evacuees and relief workers. Am Fam Physician. 2005;72:2126–34.

[bib40] Mulder AC, Pijnacker R, de Man H et al. "Sickenin' in the rain”—increased risk of gastrointestinal and respiratory infections after urban pluvial flooding in a population-based cross-sectional study in the Netherlands. BMC Infect Dis. 2019;19:377. 10.1186/s12879-019-3984-5. 31046688 PMC6498475

[bib41] Nichols GL, Freedman J, Pollock KG et al. Cyclospora infection linked to travel to Mexico, June to September 2015. Eurosurveillance. 2015;20:30048. 10.2807/1560-7917.ES.2015.20.43.30048. 26536814

[bib42] NIFC. (National Interagency Fire Center) Annual Report. 2024. https://www.nifc.gov/fire-information/statistics (Accessed Jan 2025)

[bib43] Pal A, Mann A, den Bakker HC. Analysis of microbial composition of edible insect products available for human consumption within the US using traditional microbiological methods and WGS. J Food Prot. 2024;87:100277. 10.1016/j.jfp.2024.100277. 38615992

[bib44] Romanello M, Napoli CD, Green C et al. The 2023 report of the Lancet Countdown on health and climate change: the imperative for a health-centred response in a world facing irreversible harms. Lancet. 2023;402:2346–94. 10.1016/S0140-6736(23)01859-7. 37977174 PMC7616810

[bib45] Romanello M, Walawender M, Hsu S-C et al. The 2024 report of the Lancet Countdown on health and climate change: facing record-breaking threats from delayed action. Lancet. 2024;404:1847–96. 10.1016/S0140-6736(24)01822-1. 39488222 PMC7616816

[bib46] Schwartz BS, Harris JB, Khan AI et al. Diarrheal epidemics in Dhaka, Bangladesh, during three consecutive floods: 1988, 1998, and 2004. Am J Trop Med Hyg. 2006;74:1067–73. 10.4269/ajtmh.2006.74.1067 16760521 PMC1626162

[bib47] Semenza JC, Paz S. Climate change and infectious disease in Europe: impact, projection and adaptation. Lancet Reg Health—Eur. 2021;9:100230. 10.1016/j.lanepe.2021.100230. 34664039 PMC8513157

[bib48] Sha L, Xiong YL. Plant protein-based alternatives of reconstructed meat: science, technology, and challenges. Trends Food Sci Technol. 2020;102:51–61. 10.1016/j.tifs.2020.05.022.

[bib49] Su Y, Liu A, Zhu M-J. Mapping the landscape of listeriosis outbreaks (1998–2023): trends, challenges, and regulatory responses in the United States. Trends Food Sci Technol. 2024;154:104750. 10.1016/j.tifs.2024.104750.

[bib50] Taseen S, Jawed S, Ameen AM. Cholera outbreak amidst urban flooding in Karachi: an emergency condition. Annals Med Surg. 2022;81:104365. 10.1016/j.amsu.2022.104365. PMC9450118 36091194

[bib51] US Environment Protection Agency https://www.epa.gov/climate-indicators/climate-change-indicators-sea-surface-temperature; updated June 2024; (Dec 2024, date last accessed).

[bib53] Vezzulli L, Grande C, Reid PC et al. Climate influence on *Vibrio* and associated human diseases during the past half-century in the coastal North Atlantic. Proc Natl Acad Sci. 2016;113:E5062–71. 10.1073/pnas.1609157113. 27503882 PMC5003230

[bib59_672_231325] Wade TJ, Sandhu SK, Levy D, Lee S, LeChevallier MW, Katz L and Colford JM. Did a severe flood in the Midwest cause an increase in the incidence of gastrointestinal symptoms?. Am J Epidemiol. 2004;159:398–405. 14769644 10.1093/aje/kwh050

[bib54] Wantulla M, van Loon JJA, Dicke M. Soil amendment with insect exuviae causes species-specific changes in the rhizosphere bacterial community of cabbage plants. Appl Soil Ecology. 2023;188:104854 10.1016/j.apsoil.2023.104854.

[bib55] Weinroth MD, Belk AD, Belk KE. History, development, and current status of food safety systems worldwide. Animal Front. 2018;8:9–15. 10.1093/af/vfy016. PMC6951898 32002225

[bib58] Zellweger RM, Basnyat B, Shrestha P et al. A 23-year retrospective investigation of *Salmonella* Typhi and *Salmonella* Paratyphi isolated in a tertiary Kathmandu hospital. PLoS NeglTrop Dis. 2017;11:e0006051. 10.1371/journal.pntd.0006051. PMC5720835 29176850

[bib56] Zhang W, Chen K, Zhang L et al. The impact of global warming on the signature virulence gene, thermolabile hemolysin, of *Vibrio parahaemolyticus*. Microbiol Spectr. 2023;11:e0150223. 10.1128/spectrum.01502-23. 37843303 PMC10715048

[bib57] Zingales V, Taroncher M, Martino PA et al. Climate change and effects on molds and mycotoxins. Toxins. 2022;14:445. 10.3390/toxins14070445. 35878185 PMC9319892

